# Associations of Blood Urate Level with Glycemic Status and Other Cardiometabolic Risk Factors in Middle-Aged Women

**DOI:** 10.1089/whr.2021.0029

**Published:** 2021-09-20

**Authors:** Ichiro Wakabayashi, Takashi Daimon

**Affiliations:** ^1^ Department of Environmental and Preventive Medicine and Hyogo College of Medicine, Nishinomiya, Japan.; ^2^ Department of Biostatistics, Hyogo College of Medicine, Nishinomiya, Japan.

**Keywords:** cardiovascular disease, diabetes mellitus, dyslipidemia, hypertension, urate

## Abstract

***Background:*** Hyperuricemia is a risk factor of cardiovascular disease. It remains to be elucidated how blood urate level is associated with hyperglycemia in women.

***Methods:*** The participants were 4612 middle-aged Japanese female workers. They were divided into four quartile groups by serum urate level, and cardiovascular risk factors were compared in the quartile groups.

***Results:*** With an increase of the quartile for urate, the means of waist-to-height ratio, systolic and diastolic blood pressure, log-transformed triglycerides, low-density lipoprotein (LDL) cholesterol, and cardiometabolic index (CMI) tended to be higher and high-density lipoprotein (HDL) cholesterol tended to be lower. Hemoglobin A1c was significantly higher in the 4th quartile for urate than in the 1st quartile, but this difference was not found when body mass index (BMI) was adjusted. The odds ratios versus the 1st quartile for high waist-to-height ratio, hypertension, hypertriglyceridemia, hypo-HDL cholesterolemia, hyper-LDL cholesterolemia, high CMI, and diabetes tended to be higher with an increase of the quartile. The odds ratios of the 4th versus 1st quartiles for these abnormalities except for high waist-to-height ratio and diabetes were significantly higher than the reference level even with adjustment for BMI. Hemoglobin A1c showed a weak but significant positive correlation with urate in analysis with adjustment for BMI.

***Conclusion:*** Blood urate was positively associated with adiposity, blood pressure, triglycerides, LDL cholesterol, and glycemic status and was inversely associated with HDL cholesterol in middle-aged women. The associations of urate with blood pressure, blood lipids, and glycemic status remained independent of adiposity, although being confounded by adiposity.

## Introduction

Hyperuricemia has been shown to be a risk factor of cardiovascular disease^1,2^ and to be associated with other cardiometabolic risk factors, including visceral obesity,^3^ hypertension,^4^ and dyslipidemia.^5^ However, the results of previous studies regarding the relationship of blood urate level with the risk of diabetes are conflicting. In studies in which meta-analysis was performed for both men and women, serum uric acid level was shown to be an independent risk factor for type 2 diabetes.^6,7^ In a prospective study in the United States using both male and female subjects, it was shown that urate rose before diagnosis of diabetes and then declined with duration of diabetes.^8^ Similarly, in previous cross-sectional studies conducted in the United States, United Kingdom, and Sweden, it was shown that there was a bell-shaped relationship between urate level and glycemic status both in men and in women: moderate hyperglycemia and prominent hyperglycemia are associated with higher and lower urate levels, respectively.^9,10^ On the contrary, two recent prospective studies in Sweden and China suggested a gender difference in the relationship of urate level with incident diabetes, although the results regarding gender are contradictory: high serum urate level was associated with increased risk of type 2 diabetes in men but not in women in one study^11^ and in women but not in men in the other study.^12^ In a cross-sectional study conducted in Japan, there was an inverse association between serum urate level and prevalence of diabetes in men but not in women,^13^ while in another Japanese study, serum urate level was positively associated with prevalence of newly diagnosed diabetes in women but not in men.^14^ In Japanese men, one study showed that there was no association between serum urate and fasting plasma glucose,^15^ while another study showed that serum urate level was inversely associated with hemoglobin A1c level and prevalence of diabetes.^16^ Thus, the results of previous studies regarding the relationships of urate level with prevalence of diabetes and glycemic status were inconsistent and the reason for the inconsistency is unknown. Moreover, there has been limited information on the relationship between urate level and glycemic status in women and it remains to be determined whether and how gender affects the relationship between blood urate level and glycemic status.

The purpose of this study was therefore to elucidate the relationships of blood urate level with diabetes and other cardiometabolic risk factors (visceral obesity, hypertension, and dyslipidemia) in middle-aged Japanese women. Whether and how these relationships were confounded by adiposity were also investigated by using multivariable analyses, including analysis of covariance (ANCOVA), logistic regression analysis, and linear regression analysis.

## Methods

### Participants

The participants were 4612 Japanese female workers (30–65 years) who had undergone annual health checkups from April 2005 to March 2006.^16^ The protocol of this cross-sectional study was approved by the Hyogo College of Medicine Ethics Committee (No. 3003 approved on February 25, 2020).

The status of lifestyles on alcohol consumption, cigarette smoking, regular exercise (30 minutes or longer per almost every day), and histories of illness and medication therapy for illness were surveyed by self-reported questionnaires in each participant. Participants receiving urate-lowering drugs were excluded from the subjects for analysis.

Weekly average amount of alcohol intake was reported in the questionnaires. Frequency of individual alcohol drinking was asked as “How frequently do you drink alcohol?” and was categorized as “every day” (regular drinkers), “sometimes” (occasional drinkers), and “never” (nondrinkers). Habitual smokers were categorized by daily average cigarette consumption as light smokers (20 cigarettes or less), heavy smokers (21 or more and 40 or less cigarettes), and very heavy smokers (more than 40 cigarettes) by the questionnaires. Smokers were defined as persons who had smoked cigarettes at least for the past month and for 6 months or longer.

### Measurements

At the health checkup examination, height and body weight were measured, and body mass index (BMI) was calculated as weight in kilograms divided by the square of height in meters. Waist circumference was also measured at the navel level according to the definition of the Japanese Committee for the Diagnostic Criteria of Metabolic Syndrome,^17^ and visceral obesity was evaluated by using waist-to-height ratio defined as waist circumference (cm) divided by height (cm). Blood pressure was measured at rest in a sitting position with a mercury sphygmomanometer, and Korotkoff phase V was used for definition of diastolic pressure. Fasted venous blood was collected from each participant, and concentrations of urate, triglycerides, high-density lipoprotein (HDL) cholesterol, and low-density lipoprotein (LDL) cholesterol in the serum were measured as described previously.^16^ Hemoglobin A1c concentration was evaluated by the latex cohesion method and calibrated by using a formula proposed by the Japa Diabetes Society.^18^ Cardiometabolic index (CMI), a useful index for evaluating the cardiovascular risk, was defined as the product of two ratios such as the ratio of waist circumference to height (cm/cm) and the ratio of triglycerides to HDL cholesterol (mg/dL/mg/dL).^19^

### Criteria of cardiovascular risk factors

The definition for each risk factor was as follows: visceral obesity, waist-to-height ratio ≥0.5^20^; hypertension, systolic blood pressure ≥140 mmHg and/or diastolic blood pressure ≥90 mmHg^21^; high triglycerides, triglycerides ≥150 mg/dL; low HDL cholesterol, HDL cholesterol <50 mg/dL; high LDL cholesterol, LDL cholesterol ≥140 mg/dL^22,23^; diabetes, hemoglobin A1c ≥6.5%^24^; and high CMI, ≥0.800.^19^ Participants who were receiving medication therapy for hypertension and diabetes were also regarded as those with hypertension and diabetes, respectively.

### Statistical analysis

The participants were divided into four almost equal-sized quartile groups after arranging them with serum urate levels in an ascending order. Serum urate concentrations were measured as a unit of mg/dL with one decimal place, and there was a considerable number of subjects who showed the same value of urate concentration at each quartile border. Thus, it was impossible to divide the subjects into four quartile groups consisting of completely equal numbers of subjects. The subjects were therefore divided into four quartile groups with the similar numbers of subjects: 1070 subjects in the 1st quartile, 1207 subjects in the 2nd quartile, 1157 subjects in the 3rd quartile, and 1178 subjects in the 4th quartile. Continuous variables are summarized as ranges, and means with standard deviations or medians with interquartile ranges, as appropriate. Categorical variables are summarized as frequencies (percentages). Cardiovascular risk-related variables were compared among the four quartile groups of urate by univariable and multivariable analyses as below. In the univariable analyses, the continuous variables were compared among the four groups with the use of analysis of variance (ANOVA) followed by Student's *t*-test with Bonferroni's multiplicity correction. However, triglyceride concentrations and CMI were compared with the use of the Kruskal–Wallis test followed by the Steel-Dwass test, as it is known that these do not display a normal distribution. The categorical variables were compared with the use of Pearson's chi-square test. Trends for the continuous and categorical variables were evaluated with the use of the Jonckheere-Terpstra and Cochran-Armitage tests, respectively. Correlations of the continuous variables with urate were evaluated by Pearson’ correlation coefficient. In the multivariable analyses, the continuous variables were compared among the four groups with the use of ANCOVA followed by Student's *t*-test with Bonferroni's multiplicity correction, where triglycerides and CMI were logarithmically transformed with a base of 10. In addition, the continuous variables were compared with the use of multiple linear regression analysis, where standardized partial regression parameters were estimated. The dichotomous variables were compared with the use of multiple logistic regression analysis, where the crude and adjusted odds ratios were estimated with their corresponding 95% confidence intervals. The multivariable analyses included adjustments for age, habits of smoking, alcohol drinking, and regular exercise and a medication therapy history of hypertension, dyslipidemia, or diabetes, adding BMI as needed. Pearson's correlation coefficients of BMI with urate and hemoglobin A1c were 0.293 (*p* < 0.01) and 0.278 (*p* < 0.01), respectively. Thus, there are associations between BMI and urate and between BMI and hemoglobin A1c, meaning that BMI is a confounder for the relationship between urate and hemoglobin A1c. For the purpose to clarify the relationship of urate with glycemic status, participants were arranged by their hemoglobin A1c values in an ascending order; then the participants were divided into four almost equal-sized quartile groups. Urate levels were compared in the four quartile groups for hemoglobin A1c and a hyperglycemic group of participants showing hemoglobin A1c levels of ≥5.7%^24^ and/or having a medication history of diabetes. All probability (*p*) values are two-sided. Statistical significance was defined when a *p*-value was <0.05. A computer software program (IBM SPSS Statistics for Windows, Version 25.0. Armonk, NY: IBM Corp) was used for the above statistical analyses.

## Results

### Characteristics of the participants

Table 1 shows the characteristics of the participants in this study. The mean urate level was 4.21 mg/dL and its range was from 0.40 to 9.40 mg/dL. The proportions of participants with visceral obesity (high waist-to-height ratio), hypertension, hypertriglyceridemia, hypo-HDL-cholesterolemia, hyper-LDL-cholesterolemia, and diabetes were 30.3%, 16.1%, 6.9%, 12.3%, 20.7%, and 2.1%, respectively. The proportions of participants with histories of therapy for hypertension, dyslipidemia, and diabetes were 7.7%, 4.4%, and 1.5%, respectively.

**Table 1.** Characteristics of Overall Participants

Variable	Value (number, mean, median, or frequency)
*n*	4612
Urate (mg/dL)	4.21 ± 0.94 [0.40–9.40]
Age (years)	45.7 ± 7.9 [30–65]
Smokers (%)	20.3
Drinkers (%)	
Occasional	33.0
Regular	10.1
Regular exercise (%)	5.4
Height (cm)	157.4 ± 5.5 [141.5–177.5]
Body weight (kg)	54.5 ± 9.1 [28.5–117.9]
Waist circumference (cm)	76.6 ± 9.6 [51.5–121]
BMI (kg/m^2^)	22.0 ± 3.5 [11.6–44.1]
Waist-to-height ratio	0.487 ± 0.063 [0.33–0.79]
Systolic BP (mmHg)	118.0 ± 16.9 [72–200]
Diastolic BP (mmHg)	70.7 ± 11.2 [40–128]
Triglycerides (mg/dL)	65 (47, 92) [18–1246]
HDL cholesterol (mg/dL)	65.8 ± 14.7 [25–144]
LDL cholesterol (mg/dL)	112.9 ± 29.9 [25–299]
CMI	0.472 (0.309, 0.789) [0.056–19.59]
Hemoglobin A1c (%)	5.33 ± 0.45 [4.02–13.20]
Therapy for hypertension (%)	7.7
Therapy for dyslipidemia (%)	4.4
Therapy for diabetes (%)	1.5
High waist-to-height ratio (%)	30.3
Hypertension (%)	16.1
Hypertriglyceridemia (%)	6.9
Hypo-HDL cholesterolemia (%)	12.3
Hyper-LDL cholesterolemia (%)	20.7
High CMI (%)	24.5
Diabetes (%)	2.1

Mean with standard deviation, median with interquartile range in parenthesis, or frequency (%) of each variable are shown. The range of each variable is shown in square bracket.

BMI, body mass index; BP, blood pressure; CMI, cardiometabolic index; HDL, high-density lipoprotein; LDL, low-density lipoprotein.

### Comparison of variables in the four quartile groups for urate

Table 2 shows levels of each variable in the four quartiles for urate. In the quartiles for urate, there were significant increasing trends for age, proportion of regular drinkers, and proportion of participants with a habit of regular exercise. Regarding variables related to physique, there were significant positive trends in the relationships of urate quartiles with body weight, waist circumference, BMI, and waist-to-height ratio. There were also significant positive trends in the relationships of urate quartiles with systolic and diastolic blood pressure levels. Regarding levels of blood lipids, there were significant positive trends in the relationships of urate quartiles with triglycerides, LDL cholesterol, and CMI and a significant negative trend of urate quartiles with HDL cholesterol. There were also significant positive trends in the relationships of urate quartiles with proportions of participants with hypertension, hypertriglyceridemia, hypo-HDL cholesterolemia, hyper-LDL cholesterolemia, and high CMI and proportions of participants with histories of medication therapy for hypertension, dyslipidemia, and diabetes. Although there was no significant trend in the relationship between urate quartiles and hemoglobin A1c, its level was significantly higher in the 3rd and 4th quartiles for urate than in the 1st and 2nd quartiles.

**Table 2.** Comparison of Each Variable Among the Quartile Groups for Serum Urate in Women

	1st quartile	2nd quartile	3rd quartile	4th quartile	*p* for trend
*n*	1070	1207	1157	1178	—
Urate (mg/dL)	0.4–3.5	3.6–4.1	4.2–4.7	4.8–9.4	<0.001
Age (years)	44.6 ± 7.3	45.0 ± 7.6	46.0 ± 8.1^**^^,†^	47.1 ± 8.3^**^^,††^	<0.001
Smokers (%)	21.4	18.6	20.0	21.4	0.725
Drinkers (%)					
Occasional	31.0	32.8	34.6	33.4	0.318
Regular	6.9	8.3	9.6^*^	15.5^**^^,††^	0.002
Regular exercise (%)	4.1	5.1	5.5	6.6^**^	0.009
Height (cm)	157.7 ± 5.4	157.8 ± 5.5	157.2 ± 5.4	157.0 ± 5.7^*^^,††^	0.002
Body weight (kg)	52.3 ± 7.6	52.8 ± 7.6	54.5 ± 8.6^**^^,††^	58.1 ± 11.0^**^^,††^	<0.001
Waist circumference (cm)	74.0 ± 8.2	74.8 ± 8.5	77.0 ± 9.3^**^^,††^	80.4 ± 10.6^**^^,††^	<0.001
BMI (kg/m^2^)	21.0 ± 2.9	21.2 ± 2.9	22.0 ± 3.3^**^^,††^	23.6 ± 4.2^**^^,††^	<0.001
Waist-to-height ratio	0.470 ± 0.054	0.475 ± 0.056	0.490 ± 0.061^**^^,††^	0.513 ± 0.069^**^^,††^	<0.001
Systolic BP (mmHg)	115.1 ± 15.6	115.1 ± 15.2	118.5 ± 16.8^**^^,††^	123.0 ± 18.4^**^^,††^	<0.001
Diastolic BP (mmHg)	68.5 ± 10.6	68.4 ± 10.5	71.2 ± 10.6^**^^,††^	74.5 ± 11.7^**^^,††^	<0.001
Triglycerides (mg/dL)	59.5 (45, 79)	59 (45, 83)	67 (50, 95)^**^^,††^	76 (55, 115)^**^^,††^	<0.001
HDL cholesterol (mg/dL)	66.7 ± 14.1	66.9 ± 14.2	65.9 ± 14.7	63.9 ± 15.7^**^^,††^	<0.001
LDL cholesterol (mg/dL)	106.4 ± 26.6	109.6 ± 28.2	115.6 ± 30.8^**^^,††^	119.5 ± 32.0^**^^,††^	<0.001
CMI	0.415 (0.285, 0.613)	0.419 (0.280, 0.657)	0.493 (0.323, 0.821)^**^^,††^	0.611 (0.374, 1.124)^**^^,††^	<0.001
Hemoglobin A1c (%)	5.28 ± 0.37	5.29 ± 0.40	5.35 ± 0.52^*^^,†^	5.40 ± 0.48^**^^,††^	0.348
Therapy for hypertension (%)	4.5	4.6	7.5^**^^,††^	14.1^**^^,††^	<0.001
Therapy for dyslipidemia (%)	3.0	4.0	3.7	6.6^**^^,††^	<0.001
Therapy for diabetes (%)	0.56	1.57	1.64^*^	2.04^**^	0.006
High waist-to-height ratio (%)	18.9	22.8	32.7^**^^,††^	46.0^**^^,††^	<0.001
Hypertension (%)	9.9	10.5	16.0^**^^,††^	27.7^**^^,††^	<0.001
Hypertriglyceridemia (%)	2.9	4.2	6.5^**^^,†^	13.8^**^^,††^	<0.001
Hypo-HDL cholesterolemia (%)	9.3	9.8	11.4	18.4^**^^,††^	<0.001
Hyper-LDL cholesterolemia (%)	13.3	16.8	22.8^**^^,††^	29.4^**^^,††^	<0.001
High CMI (%)	14.8	17.8	26.3^**^^,††^	38.5^**^^,††^	<0.001
Diabetes (%)	0.93	1.99	2.25^*^	3.31^**^	<0.001

Range, mean with standard deviation, median with interquartile range in parenthesis, and frequency (%) of each variable are shown. A *p*-value for the trend of relationship of quartile with each variable is also given in the table. Symbols denote significant differences from the 1st (^*^p < 0.05; ^**^p < 0.01) and the 2nd (^†^*p* < 0.05; ^††^*p* < 0.01) quartile groups.

### Comparison of adiposity index, blood pressure, blood lipids, and glycemic status in the four quartile groups of urate in multivariable analysis

Mean levels of each variable were compared in the four quartile groups of urate (Table 3). When age, histories of habitual smoking, drinking, and regular exercise and a history of medication therapy for hypertension, dyslipidemia, or diabetes were adjusted, waist-to-height ratio, systolic and diastolic blood pressure, log-transformed triglycerides, LDL cholesterol, and log-transformed CMI were significantly higher in the 3rd and 4th quartile groups of urate than in the 1st and 2nd quartiles. When BMI was additionally adjusted, these significant differences were also found in diastolic blood pressure, log-transformed triglycerides, LDL cholesterol, and CMI, but not in waist-to-height ratio and systolic blood pressure. When age, histories of habitual smoking, drinking, and regular exercise and a history of medication therapy for hypertension, dyslipidemia, or diabetes were adjusted, HDL cholesterol and hemoglobin A1c were significantly lower and higher, respectively, in the 4th quartile for urate than in the 1st quartile, while these differences were not found in the analysis with additional adjustment for BMI.

**Table 3.** Comparison of Means of Each Variable Among the Four Quartile Groups for Serum Urate in Multivariate Analysis

	1st quartile	2nd quartile	3rd quartile	4th quartile
Waist-to-height ratio				
Adjusted 1	0.471 ± 0.002	0.476 ± 0.002	0.489 ± 0.002^**^^,††^	0.511 ± 0.002^**^^,††^
Adjusted 2	0.485 ± 0.001	0.486 ± 0.001	0.488 ± 0.001	0.488 ± 0.001
Systolic BP (mmHg)				
Adjusted 1	116.3 ± 0.5	116.0 ± 0.4	118.3 ± 0.4^*^^,††^	121.2 ± 0.4^**^^,††^
Adjusted 2	117.7 ± 0.4	117.1 ± 0.4	118.2 ± 0.4	119.0 ± 0.4
Diastolic BP (mmHg)				
Adjusted 1	69.1 ± 0.3	68.9 ± 0.3	71.1 ± 0.3^**^^,††^	73.4 ± 0.3^**^^,††^
Adjusted 2	70.0 ± 0.3	69.6 ± 0.3	71.1 ± 0.3^††^	72.0 ± 0.3^**^^,††^
Log(triglycerides)				
Adjusted 1	1.79 ± 0.01	1.80 ± 0.01	1.84 ± 0.01^**^^,††^	1.90 ± 0.01^**^^,††^
Adjusted 2	1.81 ± 0.01	1.81 ± 0.01	1.84 ± 0.01^**^^,††^	1.87 ± 0.01^**^^,††^
HDL cholesterol (mg/dL)				
Adjusted 1	67.3 ± 0.4	67.1 ± 0.4	65.8 ± 0.4	63.3 ± 0.4^**^^,††^
Adjusted 2	66.1 ± 0.4	66.1 ± 0.4	65.9 ± 0.4	65.3 ± 0.4
LDL cholesterol (mg/dL)				
Adjusted 1	107.2 ± 0.9	110.2 ± 0.8	115.2 ± 0.8^**^^,††^	118.4 ± 0.8^**^^,††^
Adjusted 2	108.7 ± 0.9	111.7 ± 0.8	115.2 ± 0.8^**^^,†^	115.8 ± 0.9^**^^,††^
Log(CMI)				
Adjusted 1	−0.359 ± 0.009	−0.346 ± 0.008	−0.281 ± 0.008^**^^,††^	−0.186 ± 0.008^**^^,††^
Adjusted 2	−0.324 ± 0.008	−0.318 ± 0.007	−0.283 ± 0.007^**^^,††^	−0.244 ± 0.008^**^^,††^
Hemoglobin A1c (%)				
Adjusted 1	5.31 ± 0.01	5.30 ± 0.01	5.34 ± 0.01	5.38 ± 0.01^**^^,††^
Adjusted 2	5.33 ± 0.01	5.32 ± 0.01	5.34 ± 0.01	5.35 ± 0.01

Means with standard errors of each variable are shown. Age, histories of alcohol drinking, smoking, and regular exercise and a history of medication therapy for hypertension, dyslipidemia, or diabetes were adjusted (Adjusted 1). In addition, BMI was adjusted in another series of analyses (Adjusted 2). Symbols indicate significant differences from the 1st quartile (^*^p < 0.05; ^**^p < 0.01) and the 2nd quartile (^†^*p* < 0.05; ^††^*p* < 0.01).

### Comparison of the prevalence of abnormality in each variable in the four quartile groups of urate

Logistic regression analysis using the 1st quartile group of urate as a reference group was performed to investigate the relationships of urate with adiposity, hypertension, dyslipidemia, and diabetes (Table 4). Odds ratios for high waist-to-height ratio, hypertension, hypertriglyceridemia, hypo-HDL cholesterolemia, hyper-LDL cholesterolemia, and high CMI tended to be higher with an increase of the quartile for urate. In multivariable analysis, age, histories of habitual smoking, drinking, and regular exercise and a history of medication therapy for hypertension, dyslipidemia, or diabetes were adjusted. Both in univariable analysis and multivariable analysis, the odds ratios for high waist-to-height ratio, hypertension, hypertriglyceridemia, hypo-HDL cholesterolemia, hyper-LDL cholesterolemia, and high CMI of the 4th quartile group of urate versus the 1st quartile group were significantly higher than the reference level of 1.00. When BMI was additionally adjusted in the multivariable analysis, the odds ratios of the 4th quartile group of urate versus the 1st quartile group for hypertension, hypertriglyceridemia, hypo-HDL cholesterolemia, hyper-LDL cholesterolemia, and high CMI were still significant, but the odds ratios for high waist-to-height ratio and diabetes were not significant.

**Table 4.** Comparison of the Prevalence of Abnormality in Each Variable Among the Four Quartile Groups for Serum Urate in Women

	1st quartile	2nd quartile	3rd quartile	4th quartile
High waist-to-height ratio				
Crude	1.00	1.27 (1.03–1.56)^*^	2.09 (1.71–2.54)^**^	3.66 (3.03–4.43)^**^
Adjusted 1	1.00	1.25 (1.02–1.54)^*^	2.02 (1.65–2.46)^**^	3.68 (3.02–4.49)^**^
Adjusted 2	1.00	1.29 (0.97–1.72)	1.47 (1.11–1.95)^**^	1.28 (0.96–1.70)
Hypertension				
Crude	1.00	1.07 (0.82–1.40)	1.73 (1.34–2.23)^**^	3.48 (2.74–4.41)^**^
Adjusted 1	1.00	0.99 (0.74–1.32)	1.44 (1.10–1.88)^**^	3.03 (2.36–3.89)^**^
Adjusted 2	1.00	0.95 (0.70–1.27)	1.16 (0.87–1.53)	1.77 (1.35–2.32)^**^
Hypertriglyceridemia				
Crude	1.00	1.48 (0.94–2.33)	2.32 (1.52–3.56)^**^	5.34 (3.60–7.92)^**^
Adjusted 1	1.00	1.48 (0.93–2.34)	2.20 (1.43–3.40)^**^	5.12 (3.42–7.67)^**^
Adjusted 2		1.46 (0.91–2.32)	1.85 (1.19–2.89)^**^	3.53 (2.32–5.37)^**^
Hypo-HDL cholesterolemia				
Crude	1.00	1.05 (0.79–1.39)	1.25 (0.95–1.64)	2.19 (1.70–2.82)^**^
Adjusted 1	1.00	1.09 (0.82–1.45)	1.31 (0.99–1.73)	2.50 (1.92–3.25)^**^
Adjusted 2	1.00	1.06 (0.80–1.42)	1.12 (0.84–1.49)	1.71 (1.29–2.27)^**^
Hyper-LDL cholesterolemia				
Crude	1.00	1.32 (1.05–1.67)^*^	1.93 (1.55–2.42)^**^	2.72 (2.19–3.38)^**^
Adjusted 1	1.00	1.32 (1.04–1.69)^*^	1.78 (1.40–2.26)^**^	2.52 (2.01–3.18)^**^
Adjusted 2	1.00	1.30 (1.02–1.67)^*^	1.59 (1.25–2.03)^**^	1.77 (1.38–2.26)^**^
High CMI				
Crude	1.00	1.25 (1.00–1.57)	2.06 (1.66–2.55)^**^	3.62 (2.95–4.45)^**^
Adjusted 1	1.00	1.26 (1.00–1.58)	2.02 (1.62–2.51)^**^	3.74 (3.01–4.65)^**^
Adjusted 2	1.00	1.24 (0.97–1.57)	1.63 (1.29–2.06)^**^	2.20 (1.74–2.78)^**^
Diabetes				
Crude	1.00	2.15 (1.02–4.52)^*^	2.44 (1.17–5.08)^*^	3.63 (1.80–7.31)^**^
Adjusted 1	1.00	2.11 (0.99–4.46)	2.06 (0.97–4.35)	3.30 (1.61–6.75)^**^
Adjusted 2	1.00	2.06 (0.96–4.41)	1.55 (0.72–3.36)	1.56 (0.73–3.36)

Odds ratios with 95% confidence intervals are shown. Age, histories of alcohol drinking, smoking, and regular exercise, and a history of medication therapy for hypertension, dyslipidemia, or diabetes were adjusted (Adjusted 1). In addition, BMI was adjusted in another series of analyses (Adjusted 2). Asterisks indicate significant differences from the reference level of 1.00 (^*^p < 0.05; ^**^p < 0.01).

### Correlations of each variable with urate

Table 5 shows correlation coefficients of each variable with urate. In the univariable analysis and multivariable analysis with adjustment for age, histories of habitual smoking, drinking, and regular exercise and a history of medication therapy for hypertension, dyslipidemia, or diabetes, there were significant correlations of urate with waist-to-height ratio, systolic and diastolic blood pressure, log-transformed triglycerides, HDL cholesterol, LDL cholesterol, log-transformed CMI, and hemoglobin A1c. When BMI was added to the explanatory variables in the multivariable analysis, the correlation coefficients of systolic and diastolic blood pressure, log-transformed triglycerides, log-transformed CMI, and hemoglobin A1c with urate remained significant, but the correlation coefficients of waist-to-height ratio, HDL cholesterol, and LDL cholesterol with urate were not significant.

**Table 5.** Correlations of Each Variable with Serum Urate in Women

	Pearson's CC (Crude)	Beta (Adjusted 1)	Beta (Adjusted 2)
Waist-to-height ratio	0.271^**^	0.253^**^	0.016
Systolic BP	0.203^**^	0.128^**^	0.044^**^
Diastolic BP	0.224^**^	0.160^**^	0.078^**^
Log(triglycerides)	0.238^**^	0.212^**^	0.138^**^
HDL cholesterol	−0.073^**^	−0.102^**^	−0.013
LDL cholesterol	0.175^**^	0.085^**^	0.023
Log(CMI)	0.251^**^	0.237^**^	0.112^**^
Hemoglobin A1c	0.111^**^	0.078^**^	0.026^*^

Pearson's CC and standardized partial regression coefficients (beta) between each variable and serum urate are shown. Age, histories of alcohol drinking, smoking, and regular exercise, and a history of medication therapy for hypertension, dyslipidemia, or diabetes were adjusted (Adjusted 1). In addition, BMI was adjusted in another series of analyses (Adjusted 2). Asterisks indicate significant CC (^*^p < 0.05; ^**^p < 0.01).

CC, correlation coefficients.

### Comparison of urate levels in the four quartile groups for hemoglobin A1c and the hyperglycemic group in univariable and multivariable analyses

Table 6 shows urate levels in the four quartile groups of hemoglobin A1c and the hyperglycemic group. Both in the univariable analysis and multivariable analysis with adjustment for age, histories of smoking, alcohol drinking, and regular exercise and a history of medication therapy for diabetes, urate was significantly higher in the 3rd and 4th quartile groups and the hyperglycemic group than in the 1st and 2nd quartile groups. When BMI was also adjusted in the multivariable analysis, urate was significantly higher in the hyperglycemic group than in the 1st quartile group and was significantly higher in the 3rd and 4th quartile groups and the hyperglycemic group than in the 2nd quartile group.

**Table 6.** Comparison of Mean Urate Levels Among the Quartile Groups for Hemoglobin A1c and the Hyperglycemic Group

	Urate (mg/dL) Univariate		Urate (mg/dL) Multivariate (Adjusted 1)		Urate (mg/dL) Multivariate (Adjusted 2)	
Q1 for HbA1c	4.12 ± 0.03	4.12 ± 0.03	4.13 ± 0.03	4.12 ± 0.03	4.20 ± 0.03	4.17 ± 0.03
Q2 for HbA1c	4.08 ± 0.03	4.08 ± 0.03	4.10 ± 0.03	4.09 ± 0.03	4.13 ± 0.03	4.11 ± 0.02
Q3 for HbA1c	4.25 ± 0.03^*^^,††^	4.25 ± 0.03^*^^,††^	4.26 ± 0.03^*^^,††^	4.26 ± 0.03^**^^,††^	4.26 ± 0.03^††^	4.25 ± 0.03^††^
Q4 for HbA1c	4.41 ± 0.03^**^^,††^		4.38 ± 0.03^**^^,††^		4.27 ± 0.03^††^	
Hyperglycemia		4.62 ± 0.05^**^^,††^		4.61 ± 0.05^**^^,††^		4.44 ± 0.05^**^^,††^

Means with standard errors of serum urate levels are shown. Hemoglobin A1c (HbA1c, %): 1st quartile (Q1), 4.0–5.0; 2nd quartile (Q2), 5.1–5.3; 3rd quartile (Q3), 5.4–5.5; 4th quartile (Q4), 5.6–13.2; hyperglycemic group, 5.7 or higher. Age, histories of alcohol drinking, smoking, and regular exercise and a history of medication therapy for hypertension, dyslipidemia, or diabetes were adjusted (Adjusted 1). In addition, BMI was adjusted in another series of analyses (Adjusted 2). Symbols indicate significant differences from the 1st quartile (^*^p < 0.05; ^**^p < 0.01) and 2nd quartile (^††^*p* < 0.01) for hemoglobin A1c.

### Correlations of urate with hemoglobin A1c in subjects with hyperglycemia and subjects with diabetes

Pearson's correlation coefficients between urate and hemoglobin A1c were −0.038 (*p* = 0.428) in subjects with hyperglycemia, including diabetes (*n* = 440), and −0.074 (*p* = 0.524) in subjects with only diabetes (*n* = 76). When adjusted for age, histories of smoking, alcohol drinking, and regular exercise, a history of medication therapy for diabetes, and BMI, standardized regression coefficients of urate with hemoglobin A1c were −0.061 (*p* = 0.168) in subjects with hyperglycemia, including diabetes, and −0.164 (*p* = 0.139) in subjects with only diabetes. Thus, in univariable and multivariable analyses, there were no significant correlations between urate and hemoglobin A1c in subjects with hyperglycemia and subjects with diabetes.

## Discussion

Blood urate was shown to be associated with cardiovascular risk factors, including visceral adiposity, blood pressure, blood lipids (triglycerides, HDL cholesterol, and LDL cholesterol), and glycemic status. This is, to the best of our knowledge, the first study demonstrating that blood urate was positively associated with glycemic status and prevalence of diabetes as well as with other cardiometabolic risk factors in women and that these associations remained independent of adiposity, although being considerably confounded by adiposity. In the multivariable analysis, the associations with blood pressure, blood lipids, and glycemic status were diminished by adjustment for BMI, a general adiposity index, but remained significant after adjustment for the variables, including BMI: the odds ratios for hypertension and dyslipidemia of the highest versus lowest quartiles for urate were significantly higher than the reference level (Table 4) and urate was significantly correlated with hemoglobin A1c (Table 5). In addition, the results of analysis using the quartile groups of hemoglobin A1c (Table 6) suggested a BMI-independent association between urate and glycemic status. Therefore, the associations of urate with blood pressure, blood lipids, and glycemic status in middle-aged Japanese women were independent of adiposity. Moreover, the relationships of urate with visceral adiposity, blood pressure, blood lipids, and glycemic status were not changed in multivariable analysis with adjustment for lifestyles, including smoking, alcohol drinking, and regular exercise. Thus, the associations of urate with the cardiovascular risk factors are also independent of the above lifestyles. There was also a relatively strong positive association between urate and CMI, a recently proposed lipid-related index that is defined by using triglycerides-to-HDL cholesterol ratio and waist-to-height ratio.^19^ The associations of urate with adiposity, hypertension, dyslipidemia, and diabetes suggest that there is a common etiology for hyperuricemia and the other cardiometabolic risk factors, in which dietary and nutritional factors may be involved because the associations were potently confounded by BMI.

In previous studies, blood urate level was inversely associated with hemoglobin A1c and prevalence of diabetes in Japanese men.^13,16^ In the present study, urate level was positively associated with hemoglobin A1c and prevalence of diabetes in middle-aged Japanese women, in agreement with the finding in a previous study in Japan that serum urate was positively associated with prevalence of impaired fasting glucose and diabetes in logistic regression analysis in women, although no association between them was found in men.^14^ Thus, there was a clear gender-related difference in the relationship between urate and glycemic status. It is known that blood urate level is lower in women than in men.^25^ One possible explanation for the inverse association between blood urate and glycemic status in men is an increasing action of insulin on renal tubular reabsorption of urate^26,27^: in patients with diabetes, urinary excretion of urate is increased by less tubular reabsorption of urate due to insulin deficiency, resulting in lower blood urate levels. In the present study, urate was higher in the group of subjects with hyperglycemia than in the lower quartile groups for hemoglobin A1c in women (Table 6). Thus, we confirmed a positive association between urate and glycemic status in overall subjects. However, there was no significant correlation between urate and hemoglobin A1c in subjects with diabetes or hyperglycemia, although their population sizes were small. When blood urate level is lower, urinary excretion level of urate is also lower and is thus expected to be less affected by insulin. Therefore, lower blood urate levels in women than in men might partly explain the gender-related difference in the relationship between urate level and glycemic status. Hyperuricemia has been suggested to inhibit pancreatic *β* cell function by causing oxidative stress.^28^ Interestingly, serum urate level has been reported to show a significant inverse correlation with insulin secretion in women but not in men.^29^ Thus, there is a possibility of a gender difference in oxidative stress-induced *β* cell dysfunction. Further studies are needed to clarify the reason for the gender difference in the relationship of blood urate level with glycemic status and diabetes.

There are limitations of this study. The number of subjects with diabetes was not large enough for analyzing their data separately in detail, and only linear regression analysis was performed to investigate the relationship between urate and glycemic status in subjects with hyperglycemia or diabetes. Diabetes was diagnosed by using result of hemoglobin A1c level and a history of medication therapy for diabetes in the questionnaires, and thus, there is a possibility of informational bias. The type of diabetes was not identified for each subject; however, the type for a majority of the subjects diagnosed as diabetes in the present study is expected to be type 2 because the prevalence of type 2 diabetes has been reported to be much higher than that of type 1 diabetes in middle-aged Japanese.^30,31^ Age, histories of lifestyles, including smoking, alcohol drinking, and regular exercise, a history of medication therapy for hypertension, dyslipidemia, or diabetes, and BMI were adjusted in the multivariable analyses. However, there are possibilities of confounding by some other factors, including nutrition, food intake, socioeconomic factors (*e.g.*, education, occupation, and income), and status of menopause, for which information was not available in the database used in this study. In addition, information on category of alcohol beverage, which affects urate level,^32^ was not available in the present study. The subjects were all Japanese, and serum urate level was positively associated with hemoglobin A1c level in the present study. There is an ethnic difference in the relationship between blood urate level and glycemic status in women since a bell-shaped relationship between them was shown in studies conducted in the United States, United Kingdom, and Sweden.^9,10^ In addition, there may be an ethnic difference in the relationship between urate level and incidence of diabetes: In women, a high serum urate level was associated with the risk of type 2 diabetes in a study conducted in China,^12^ but not in a study conducted in Sweden.^11^ Thus, further studies are needed to clarify the reasons for the ethnic differences in the relationships of blood urate level with glycemic status and risk of diabetes in women. Since adiposity has been shown to be affected by ethnicity^33^ and adiposity considerably confounded the relationship between urate level and glycemic status in the present study, ethnic difference in adiposity might explain the controversy of findings in previous studies on the relationship between serum urate level and risk of diabetes. Finally, since the design of this study is cross-sectional, further studies with a prospective design are needed to investigate causal relationships of urate with cardiovascular risk factors.

## Conclusions

Blood urate showed positive associations with adiposity, blood pressure, triglycerides, LDL cholesterol, and glycemic status and an inverse association with HDL cholesterol in middle-aged Japanese women. The associations of urate with blood pressure, blood lipids, and glycemic status remained independent of adiposity, although these associations were partly confounded by adiposity.

## Abbreviations Used

ANCOVA: analysis of covariance
ANOVA: analysis of variance
BMI: body mass index
CC: correlation coefficients
CMI: cardiometabolic index
HDL: high-density lipoprotein
LDL: low-density lipoprotein
